# Autism spectrum disorder, very-early onset schizophrenia, and child disintegrative disorder: the challenge of diagnosis. A case-report study

**DOI:** 10.3389/fpsyt.2023.1212687

**Published:** 2023-07-28

**Authors:** Michelangelo Di Luzio, Silvia Guerrera, Maria Pontillo, Maria Rosaria Lala, Laura Casula, Giovanni Valeri, Stefano Vicari

**Affiliations:** ^1^Child and Adolescent Neuropsychiatry Unit, Bambino Gesù Children’s Hospital, IRCCS, Rome, Italy; ^2^Life Sciences and Public Health Department, Catholic University, Rome, Italy

**Keywords:** child disintegrative disorder, autism spectrum disorder, very-early onset schizophrenia, neurodevelopment, late regression, case report

## Abstract

**Background:**

Autism spectrum disorder (ASD) in the Diagnostic and Statistical Manual of Mental Disorders Fifth Edition (DSM-5) contains several disorders previously present as distinct diagnoses in the DSM Revised Fourth Edition (DSM-IV-TR). These include child disintegrative disorder (CDD). The latter presents typical features, such as a late regression of developmental acquisitions. However, it also shows symptoms similar to ASD, and psychotic symptoms, such as very-early onset schizophrenia (VEOS), are described in the literature.

**Case report:**

In this case report we deepen the case of P., a child who presents a late regression, at 7 years old, associated with psychotic symptoms in the absence of organic alterations. The child was treated with antipsychotic drug therapy and cognitive behavioral therapy. P. was diagnosed with ASD with acute and late regression associated with psychotic symptoms. During the follow-up, there was a gradual improvement in the clinical conditions. Improvements were possible due to therapeutic intervention (pharmacological and psychotherapeutic) and/or the natural course of the disorder.

**Conclusion:**

The diagnostic difficulty of this case reflects a clinical complexity in which it is not easy to distinguish between neurodevelopmental and psychiatric aspects. Clinical cases such as that of P. emphasize the theme of the neurodevelopment continuum model in which neurodevelopmental and psychiatric disturbances can be considered within a pattern of pathological continuity.

## 1. Introduction

The DSM-5 changed the concept of autism from a multicategorical diagnostic system to a single diagnosis based on multiple dimensions. This transition removed diagnostic subcategories within the pervasive developmental disorders (PDD) classification (autistic disorder, Asperger’s disorder, pervasive developmental disorder not otherwise specified, Rett’s disorder, and childhood disintegrative disorder); hence, the dimensional diagnosis of autism spectrum disorder (ASD) defined as a neurodevelopmental disorder characterized by impairment in develop of sociolinguistic communication and behavioral skills with associated restricted interests and repetitive behaviors (1). Atypias can be observed early in the development of many children with ASD (1).

In 1908, a group of children were observed exhibiting typical development until the ages of 3 or 4, followed by a sudden and severe decline in cognition and language. This regression was often accompanied by mood dysregulation (2). Heller initially defined this condition as “infantile dementia.” Subsequent changes in nomenclature included “disintegrative psychosis” and “pervasive developmental disorder with childhood onset,” as described in the International Classification of Diseases, Ninth Revision (ICD-9) (3) and the Diagnostic and Statistical Manual of Mental Disorders, Third Edition (DSM-III) (4). In the tenth edition of ICD (ICD-10) (5) and the Revised Fourth Edition of the DSM (DSM-IV-TR) (6), the disorder was referred to as disintegrative childhood disorder (CDD). The inclusion criteria for CDD encompass the following: a period of apparently normal development lasting more than 2 years (including age-appropriate verbal and non-verbal communication, social relationships, play, and adaptive behavior); and clinically significant loss of previously acquired skills (between the ages of 2 and 10 years) in at least two areas, such as expressive or receptive language, social skills or adaptive behavior, bowel or bladder control, play, and motor skills. Additionally, abnormalities in functioning must be present in at least two of the following areas: qualitative impairments in social interaction (e.g., impairments in non-verbal behaviors, failure to develop peer relationships, and a lack of social or emotional reciprocity); qualitative impairments in communication (e.g., delay or absence of spoken language, inability to initiate or sustain a conversation, stereotyped and repetitive use of language, and a lack of varied make-believe play); and restricted, repetitive, and stereotyped patterns of behavior, interests, and activities, including motor stereotypies and mannerisms; and a general loss of interest in objects and the environment. Furthermore, the disturbance cannot be better explained by another specific pervasive developmental disorder, schizophrenia, acquired aphasia with epilepsy, elective mutism, or Rett syndrome (6). Over time, several factors have contributed to conflicting conclusions about the nosological validity of CDD, such as its low prevalence (7), the absence of large case–control studies, and the limited differentiation of clinical criteria between CDD and ASD (8). Consequently, owing to ongoing debate and limited literature, CDD was excluded as a distinct diagnostic category in the DSM-5 (2, 8). Various arguments have been presented in favor of validating CDD as a single diagnostic category, including greater male prevalence, onset following a typical developmental period, more pronounced and rapid regression compared with ASD, more significant cognitive impairment than ASD, frequent loss of motor skills and continence (which are rare in ASD), higher frequency of seizures than ASD, and a generally poorer prognosis for individuals with CDD compared with ASD (9).

Similar to autism, in DSM-5, schizophrenia is dimensionally reorganized in a schizophrenia spectrum disorder (SSD). Schizophrenia is a rare neuropsychiatric disorder in childhood, with a prevalence of 0.05% in young people before 13 years of age, and 2% of schizophrenia adult-onset patients report that their psychotic symptoms began before the age of 13 (10). The National Institute of Mental Health (NIMH) has described very-early-onset schizophrenia (VEOS) as a kind of schizophrenia with an onset before the age of 13. VEOS tends to be a more serious disease (10, 11) than adult-onset schizophrenia, presenting after 18 years of age (10). VEOS patients show a more premorbid neurodevelopment difficulty load, in particular with respect to early and adult-onset schizophrenia (11). Genetic conditions, such as 22q11 deletion syndrome, may lead to a greater likelihood of being identified with psychosis (12).

Scientific literature has long since highlighted the frequent psychiatric comorbidity in children and adolescents with ASD. In particular, SSD and other psychotic disorders ranged from 4 to 67% (10, 13–15). In addition to the strong comorbidity, some genetic variants would appear to be common to both disorders (16).

CDD shows to share common features both with ASD and VEOS but at the same time shows important differences with these disorders. CDD and ASD have an impairment in the sociolinguistic communication dimension. Poor investment in social communication and a social withdrawal have been considered overlapping symptoms between ASD and the prodromes of schizophrenia. According to DSM IV-TR criteria for CDD, a decline in acquired developmental skills is expected, with a rapid course between 2 and 10 years of age distinguishing it from ASD cases with a more gradual regression before the age of 2. Moreover, CDD onset is different from the onset in VEOS, as it is generally insidious and occurs after 10 years of age (17, 18). In addition, VEOS does not show a regression in development skills as the main symptomatology, but more a social withdrawal associated with negative symptoms (blurred effects, apathy, and anhedonia) (19). In a recent case study, Di Vara et al. (13), focused on the later onset of loss of previously acquired skills in sociality, communication, bowel/bladder control, and general impairment as more indicative of CDD than ASD or VEOS features. On the other hand, in CDD, the presence of affective symptoms, feelings of oddity, and/or the presence of hallucinations were described in the literature in association with regression (2, 14, 19–26). CDD shares these features with SSD but not with ASD. Finally, CDD and ASD show a high frequency in males, and VEOS shows a similar frequency in males and females (differently from adolescent and adult schizophrenia, which are much more frequent in males) (20, 27, 28). In Table 1, the main differences between CDD, ASD, and VEOS, in terms of onset, gender, and symptomatology, are summarized.

**Table 1:** Differential diagnosis among disorders.

	ASD	CDD	VEOS
Onset	Gradual regression before 2 years	Acute regression after 2 years and before 10 years	Most common, insidious onset after 10 years and before 13 years
Gender	M	M	M ≅ F
Symptoms	Atypical social-communicative development and restricted interests and repetitive behavior	Skills loss in language, social interactions, adaptative behavior, play, motricity, and bladder/bowel control Hallucinations and bizarre thought contents and speech or behaviors with disorganization are described in the literature	“Positive” as hallucinations or delusions “Negative” as social retreat Disorganized behavior or speech

ASD, autism spectrum disorder; CDD, child disintegrative disorder; F, female; M, male; VEOS, very early-onset schizophrenia.

These findings support the neurodevelopmental continuum model, which holds that childhood neurodevelopmental difficulties and psychiatric disorders, in particular those with childhood onset (e.g., VEOS), belong to an etiological and neurodevelopmental continuum, as also shown by genetic studies, and should not be interpreted as different entities (10, 15, 16, 19, 29–31).

In light of the evidence, we report the case of an 8-year-old Caucasian child with a history of neurodevelopment difficulties (speech delay and primary encopresis until 5 years of age) who presented in June 2021, at the age of 7, a sudden regression of neurodevelopmental skills, adaptive abilities, bladder control, and loss of speech associated with hallucinations and unusual thought contents without any apparent organic causes.

## 2. Case report

P. is the only child of a couple with a referred negative familial history for neuropsychiatric disorders and whose mother’s pregnancy concluded at 37 gestational weeks by cesarean section due to an abnormal intrauterine position. The couple denied any suffering at birth and reported a birth weight of 3,700 g. Autonomous walking was reported to be reached at approximately 16 months. A delay in language skills was reported that required speech therapy with partial benefit and persistent phonetic-phonological errors. These difficulties in expressive language have been reported to cause a state of social anxiety in the patient. Sphincterial control was also reached late, with reported encopresis until the age of 5 years. Abnormalities in bladder control were not reported. In the remote pathological history, a *Salmonella Typhi* infection at age 3 years required hospitalization. Simple motor (involving the neck) and vocal (cough) tics were reported previously, which spontaneously remitted.

The onset of symptoms was reported at 7 years of age, at the end of May 2021. P. showed social closing and a tendency for muteness, which the parents attributed to a stressful period (returning to school after Covid-19 lockdown, bullying episodes, and the use of a video game featuring adult content). Two weeks later abnormal thought contents appeared as thought insertion, with some features of auditory and visual hallucinatory symptoms (he claimed his mother was speaking in his head and joking with him, and he reported he was frightened by some monsters on his village roads and square). These symptoms were self-limited and lasted a few days. He also reported body-related symptoms, such as aspecific abdominal pain and the presence of a “thing in abdomen which might be removed.” General conditions were normal at a pediatric check-up.

In September 2021, after the summer holidays, symptoms quickly worsened with the development of a passive attitude, an absence of social interaction, a loss of speech, repetitive stereotyped movements, a tendency to orally explore objects, emotional and behavioral dysregulation and episodes of disruptive rage at school, anxiety, and fear. Positive affectivity was always been reported. A neuropsychiatric control was performed in another hospital in his region of residence, in October 2021, completed by brain magnetic resonance imaging (MRI) and an electroencephalogram (EEG) while he slept, which report normal findings. Antipsychotic therapy was started with risperidone associated with cognitive behavioral therapy (CBT). Urinary incontinence, with regression in bladder control, occurred between November 2021 and May 2022. However, incontinence is described in the literature as a side effect of risperidone (32), although in this case incontinence disappeared despite the continuation of risperidone at the same dosage.

He first visited the Child and Adolescent Psychiatry Unit, Bambino Gesù Children’s Hospital, IRCCS, in July 2022. During Summer 2022, there was an improvement in the clinical status, with a slight social opening and the demise of disruptive rage; therefore, a decalage of risperidone was started until 0.75 mg/die.

We have known P. since September 2022, when he attended the Child and Adolescent Psychiatry Unit, Bambino Gesù Children’s Hospital, IRCCS, for hospital day care. On this occasion, a psychiatric visit took place. P. presented with a passive attitude and loss of speech and was disorganized when exploring the surrounding environment and prone to putting objects in his mouth as a sort of oral exploration and display of disinterest in social interaction. There was no evidence of a deviation of mood or apparent signs of a manic, depressive, or dysphoric mood. His behavior showed no depressive or manic features (e.g., slow or accelerated movements) but was rather incongruous and disorganized, with fixed, non-contextual, smiling, and motor stereotypes visible. Eye contact was present but not well-modulated for social communication. At the end of hospital day care in September 2022, risperidone was replaced with olanzapine at a maximum dose of 5 mg/die. Additionally, he received an indication to intensify CBT and add speech therapy (both twice a week).

At the follow-up control with a second psychiatric visit in November 2022, he appeared to show better compliance toward the clinicians but with little improvement in social and communicative opening; however, non-contextual smiling, disorganized behavior, and a less severe mutism persisted (he said only a few words upon request). The contact of gaze was always present, and the affectivity was preserved. Play persisted in a stereotypical way, as did the oral exploration of objects of interest and possible abdominal pain complaints experienced with anxiety; however, motor stereotypes were reduced. Parents reported a sporadic episode of possible visual hallucinations in which P. was scared by the presence of an “unknown man” in his house. We conducted a comprehensive medical assessment to exclude neurological, infectious, autoimmune, or metabolic causes, as indicated in the scientific literature for the onset of a serious alteration of behavior with atypical manifestations and possible psychotic symptoms in childhood (19, 33). The assessment was conducted after hospitalization and further instances of hospital day care in January and February, 2023. On this occasion, brain MRI and EEG were repeated, and metabolic screening and spinal tap in search of inflammatory indexes, autoimmunity, and neurotropic viruses were performed. Moreover, to further explore neurodevelopmental, psychiatric, psychosocial, and familial aspects, a comprehensive assessment combining neuropsychological and psychopathological evaluations, along with social and familial observations, was conducted. The results of the multidisciplinary evaluation are reported below:

### 2.1. Neurophysiological and neuroradiological assessment

An EEG was performed, recording awake activity, with the presence of spindle-like short bursts of theta rhythm on the frontal regions, of non-specific significance. An EEG was repeated with non-specific findings of a diffuse fast rhythm. Brain MRI (T1 and T2 weighted), diffusion-weighted imaging (DWI), and magnetization-prepared rapid acquisition gradient echo (MPRAGE) were conducted. The MRI showed no altered cerebral tissue signal, no altered diffusivity at DWI, normal morpho-volumetry of the ventricular system, and a median line structure in axis. In general, no pathological findings were found with the EEG and MRI.

### 2.2. Biochemical and instrumental assessment

A metabolic screening was performed with an indication to perform in-depth metabolic blood and genetic testing. A lumbar puncture and serum analysis did not show the presence of neuroinflammation of any origin (autoimmune, metabolic, or infective). There was an isolated presence of anti-cardiolipine IgM antibodies of non-specific significance and high levels of glycated hemoglobin without diabetes in his serum. Metabolic screening was negative. The genetic visit did not find any signs or symptoms that suggested a genetic disease. Screening for celiac disease was performed and produced a negative result. As there was a referred history of constipation, to exclude an organic cause for abdominal pain, an abdomen ultrasound was undertaken and showed no pathological findings.

### 2.3. Neuropsychological and psychopathological assessment

The Leiter International Performance Scale, Third Edition (Leiter-3) (31), was used for the neurodevelopmental assessment. Leiter-3 is a tool for assessing non-verbal cognitive level, memory, and attention skills, which was designed to be administered to individuals without language difficulties and provides a non-verbal intelligence quotient (NVIQ). In our case, a non-verbal NVIQ of 74 was obtained (borderline score). The adaptive behavior profile was assessed using the Adaptive Behavior Assessment System, Second Edition (ABAS-II) (34). The TABAS-II is a parent-report questionnaire that measures a child’s skills related to development, behavior, and cognitive abilities, from which a below-average General Adaptive Composite emerged. Regarding autism symptomatology, we used the two main “gold standard” tools, performed by a well-trained clinician. The presence of autism symptoms in the past and in the present were deepened through The Autism Diagnostic Interview Revised (ADI-R) (35) and the Autism Diagnostic Observation Schedule-Second Edition (ADOS-2) (36). The ADI-R is a structured clinical caregiver interview that provides historical information, mainly for the time period between 4 and 5 years of age, as well as current information focusing on ASD-related symptoms. The ADI-R results did not suggest ASD during preschool age. However, from the age of 7, the ADI-R scores exceeded the cut-off for all subscales. Indeed, parents referred to the presence of a significant regression in language abilities, social interaction skills, and sphincter control. Qualitative abnormalities in reciprocal social interaction, stereotyped language, atypical oral object exploration, and hand stereotypies were reported. Current ASD symptoms were also confirmed through the administration of ADOS-2, a semi-structured direct assessment of communication, social interaction, and play with, or the imaginative use of, materials for individuals with suspected autism. Module 1, used for children who do not use phrase speech consistently, was performed. The ADOS 2 Calibrated Severity Score result suggested a moderate level of ASD symptom severity. Regarding psychopathological assessment, we used the Schedule for Affective Disorders and Schizophrenia for School-Aged Children Present and Lifetime Version DSM-5 (K-SADS-PL) (37), a semi-structured interview based on DSM-5 criteria that investigates the present or life-time occurrence of psychiatric symptoms in adolescent or child subjects. The K-SADS-PL interview was administered only to parents when there was a lack of cooperation from their child. The parents exposed the child’s social fears and phobias, such as a fear of sleeping alone, people dying, falling, and being hurt, since the age of 3 or 4 years old. Parents reported several possible environmental stressors that may have contributed to the onset of symptomatology. These included recent exposure to a traumatic video game during playtime, characterized by expressing the concept of death and past experiences of bullying during early childhood, where schoolmates used to tease him with verbal jokes, and the initial years of primary school, as well as the impact of the SARS-CoV-2 pandemic and subsequent lockdown, during which he spent his time in social isolation playing video games. From the age of 7, parents reported the presence of apathy, loss of interest in the environment, limited use of facial expressions, social withdrawal, some bizarre thoughts such as a “fear that his mother might play tricks on him,” visual hallucinations of “frightening monsters standing on the village square,” enuresis episodes, and the presence of some vocal tics and simple motor tics involving the eyes, arms, and shoulders. During the interview, there were no suggestions of symptoms related to major depressive disorder or manic or hypomanic episodes. P. did not manifest sadness, negative thoughts, inappetence, or self-harm. On the other hand, he never showed grandiosity, dangerous behavior, or a reduced need for sleep. Furthermore, the description of the symptomatology did not align with a relapsing–remitting pattern as with mood disorders but instead demonstrated a continuous and progressive decline in functional abilities without any signs of recovery to premorbid functioning. There were no clear symptoms indicative of post-traumatic stress disorder or adjustment disorder that emerged during the interview. The Children’s Global Assessment Scale (CGAS), which evaluates the influence of psychiatric symptoms on the subject’s functioning, showed a moderate degree of impairment of functioning in most social areas. Table 2 summarizes the main assessment findings.

**Table 2:** Assessment findings.

Neurophysiological and neuroradiological assessment	Biochemical and instrumental assessment	Neuropsychological and psychopathological assessment
EEG in awake activity: (Nov. 2022) Presence of spindle-like short bursts of theta rhythm on the frontal regions, of non-specific significance (Jan. 2023) Non-specific findings of diffuse fast rhythms.	Serum analysis: Normal findings, no evidence of autoimmunity except for the presence of anti-cardiolipine IgM antibodies of non-specific significance and high levels of glycated hemoglobin without diabetes	Leiter-3: NVIQ of 74 (borderline score)
MRI (T1 e T2, FLAIR, DWI e MPRAGE): No altered signal of cerebral tissues, no altered diffusivity at DWI. Normal morfovolumetry of the ventricular system Median line structure in axis	Metabolic screening: no evidence of metabolic disease	ABAS-II: below-average General Adaptive Composite (GAC)
	Lumbar puncture: no evidence of neuroinflammation	ADI-R: not suggestive for ASD at preschool age Scores exceed the cutoff for all its subscales after the age of 7
	Celiac screening: no pathological findings	ADOS-2, module 1: Calibrated Severity Score (CSS) result was suggestive of a moderate level of ASD symptom severity
	Eco-abdomen: no pathological findings	K-SADS-PL (only parents): fear and phobias since 3–4 years of age From age of 7: feelings of apathy, loss of interest in the environment, limited use of facial expressions, social withdrawal, some bizarre thoughts such as “fear that his mother might play tricks on him,” visual hallucinations of “frightening monsters standing on the village square,” enuresis episodes, and the presence of some vocal tics and simple motor tics
		CGAS: a moderate degree of impairment in functioning in most social areas
		Psychosocial assessment and familial observation: no evidence of trauma emerged

ABAS-II, Adaptive Behavior Assessment System, Second; ADI-R, Autism Diagnostic Interview Revised; ADOS-2, Autism Diagnostic Observation Schedule-Second Edition; CGAS, Children’s Global Assessment Scale; DWI, diffusion weighted imaging; EEG, electroencephalogram; Leiter-3, Leiter International Performance Scale, Third Edition; Jan., January; Nov., November; MPRAGE, magnetization-prepared rapid acquisition gradient echo; MRI, magnetic resonance imaging; NVIQ, non-verbal intelligence quotient; K-SADS-PL, Schedule for Affective Disorders and Schizophrenia for School-Aged Children Present and Lifetime Version DSM-5.

To rule out any potential trauma or familial abuse as a cause, we deepened psychosocial aspects through anamnestic data and conducted a thorough observation of the family context, involving experts in relational systemic psychology. The child’s father was a freelance worker, and his mother was a housewife. There were no economic problems and the family appeared to belong to the middle class. There were no signs of familial psychological or physical abuse. Both parents demonstrated a high level of attentiveness and sensitivity towards their son’s needs and emotions. P.’s family had the emotional and material support of the grandparents, who played an active and affectionate role in the patient’s daily life.

In conclusion, a descriptive diagnosis of autism spectrum disorder with an acute and late regression in association with psychotic symptoms and a loss of primary autonomies was performed for P. This description seems similar to the inclusion criteria for CDD and more complete than the simple diagnosis of ASD or VEOS. Notably, no loss of acquired social-relational communication skills emerged in the child’s developmental history until the age of 7. In addition, during the first meeting, the parents showed videotapes of the child at age 4 that featured communicative initiative, social reciprocity, and shared play with peers and adults. The child also showed a social smile by using verbal language to relate to peers. Finally, the ADI-R results did not suggest preschool ASD, as previously reported. In terms of psychotic symptoms, bizarre thoughts and visual hallucinations were not associated with a history of hyperreactivity or hyporeactivity to sensory input, either before or after the individual episodes. Therefore, we have framed these individual episodes as possible hallucinations associated with abnormal thought contents, as reported in the K-SADS-PL interview. However, the challenge lies in elucidating the nature of such symptoms definitively, as the absence of language and socio-communicative impairments hinder the exploration of thought content and the presence of hallucinations. Furthermore, the potential psychotic symptoms, particularly unusual thought contents, manifested throughout different phases of the disorder albeit not in a continuous manner. Moreover, the onset of psychotic symptoms is very rare before the age of 13 and atypical before 10 years old (17, 18). A diagnosis of an affective disorder was excluded based on the findings from the K-SADS-PL assessment, clinical observation, and anamnesis; in fact, there were no clear indications of mood disturbances or a return to the premorbid level of functioning. In particular, symptoms such as apathy, reduced interest in the environment, reduced use of facial expressions, and social withdrawal seem to belong more to a negative symptomatology (i.e., blunted affectivity) rather than a depressive symptomatology. Moreover, anxiety seems to be a consequence of the remaining symptomatology rather than the underlying cause. The psychosocial and familial assessment did not reveal any specific traumatic events or child abuse, and the previously mentioned potential stressors (bullying, video game, and COVID-19 lockdown) did not account for the severity and persistence of the symptoms observed. Furthermore, the K-SADS-PL assessment did not yield any suggestive elements for a diagnosis of post-traumatic stress disorder or adjustment disorder. A longitudinal investigation of the case may provide further answers. However, we must consider what previously explained that CDD diagnosis, despite being suggested as a separate entity, is placed in a continuum with the other two conditions.

The therapies adopted in these cases are both pharmacological and psychotherapeutic or educational. In our case, we opted for a CBT intervention for socio-communicative and behavioral regression and the use of antipsychotic treatment for psychotic symptomatology and behavioral dysregulation. Risperidone was suspended for partial effectiveness and suspected sedation and replaced with olanzapine, as the latter has been used for hyperactivity and behavioral problems in childhood disintegrative disorder and is considered a good option for second-line therapy in VEOS after risperidone to control psychotic symptoms (23, 38–42). P. showed good compliance with the assessment and the therapies administrated. The improvements achieved have made it possible to continue with this type of therapy.

**Figure 1:**
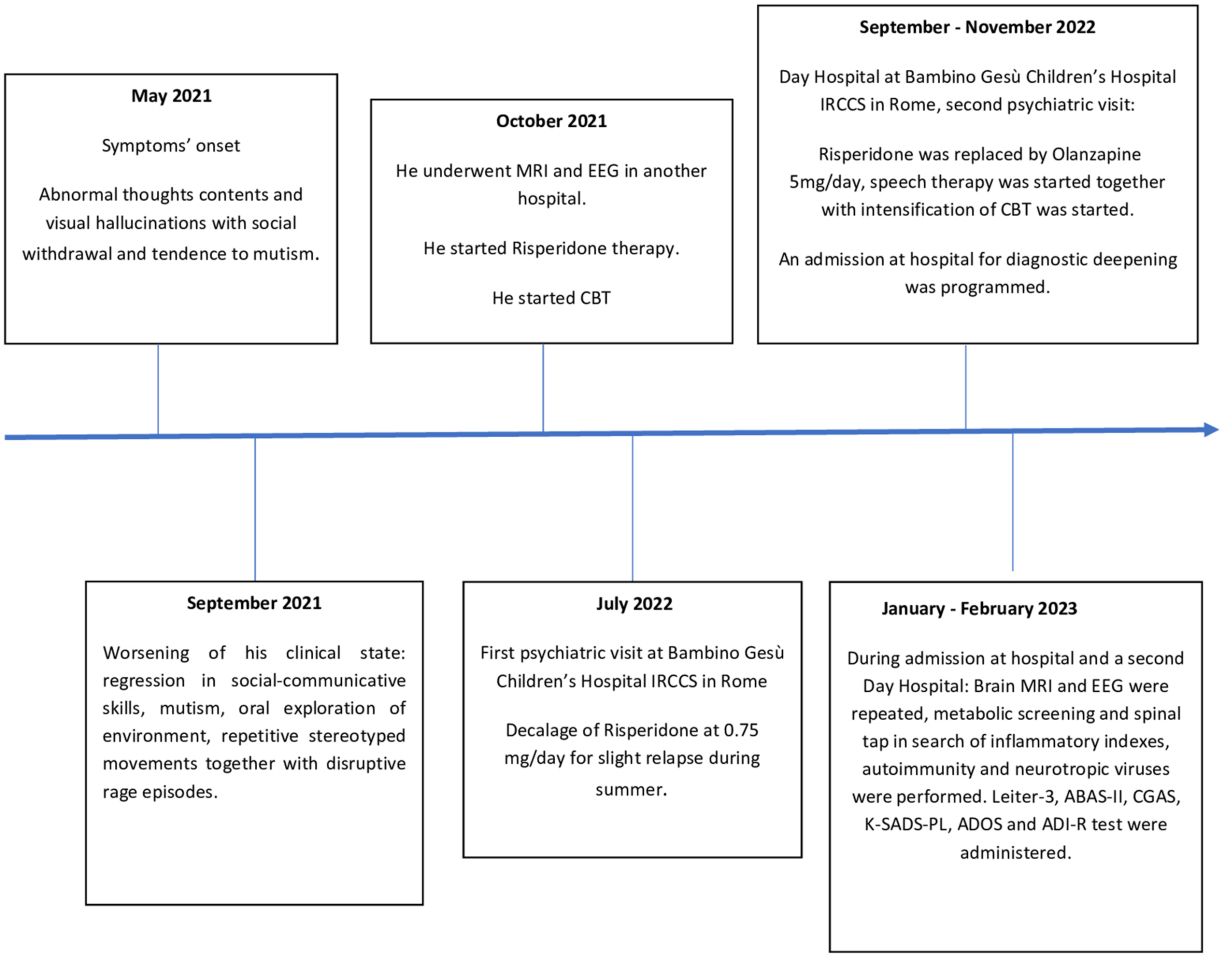
Timeline of assesments. ABAS-II, Adaptive Behavior Assessment System, Second; ADI-R, Autism Diagnostic Interview Revised; ADOS-2, Autism Diagnostic Observation Schedule-Second Edition; CBT, cognitive behavioral therapy; CGAS, Children’s Global Assessment Scale; EEG, electroencephalogram; Leiter-3, Leiter International Performance Scale, Third Edition; MRI, magnetic resonance imaging; K-SADS-PL, Schedule for Affective Disorders and Schizophrenia for School-Aged Children Present and Lifetime Version DSM-5.

From November 2022 (18 months after the onset of symptoms) until the current time, P. showed a slight and slow but continuous improvement in speech and social interaction with fewer stereotypic movements, diminished anxiety, and sporadic psychotic symptoms. However, up until now, P. has not exhibited a return to a premorbid level of functioning. Our research group continues to monitor P. with follow-up appointments approximately every 3 months. A summarized timeline of the assessments is shown in Figure 1.

## 3. Discussion

The case report primarily focuses on the process of differential diagnosis and then on results and reflections derived from this process. To exclude potential neurological, infectious, autoimmune, or metabolic causes, several assessments were necessary. It is well-documented in the scientific literature that when there is a significant change in behavior with potential atypical psychotic manifestations it is important to exclude any other non-psychiatric causes (e.g., encephalitis or a brain tumor) (19, 33). Therefore, the patient underwent brain MRI, EEG, metabolic screening, and a spinal tap to investigate inflammatory markers, autoimmunity, and neurotropic viruses. These screenings yielded negative results, ruling out other medical causes for the patient’s symptomatology. To delve deeper into the neuropsychiatric and psychosocial aspects, comprehensive neuropsychological and psychopathological evaluations were conducted, along with social and familial observations. This assessment was necessary to evaluate the presence of neurodevelopmental disorders such as ASD, psychiatric disorders including mood disorders and SSD, and psychosocial causes such as trauma or child abuse episodes. We excluded the presence of mood disorders, anxiety disorders, post-traumatic stress disorder, and adjustment disorder.

The first recommended intervention in childhood, especially when there is suspicion of a neurodevelopmental disorder such as ASD, is cognitive-behavioral psychotherapy. However, owing to the severity of the symptoms, particularly the psychotic symptoms and behavioral dysregulation, we opted for the integration of pharmacotherapy into the treatment plan. Research has shown that an integrated approach combining pharmacological and psychotherapeutic interventions is more effective than using either intervention alone (43, 44).

As our evaluations did not indicate the presence of traumatic or psychosocial causes, we did not deem it necessary to involve other interventions, such as social services evaluations or family psychotherapy. Additionally, as no indications of affective disorders emerged from the assessment, we did not use antidepressant or mood stabilizer medications.

The case of P. allows us to approach the issue of the differential diagnosis between CDD, VEOS, and ASD. This clinical presentation, like other similar (13, 22, 23, 25) presents many difficulties with regard to diagnosis and thus treatment. Albeit it shows a symptomatology of impaired communication and social interaction as framed in ASD. However, it appears clear that the child has achieved up to the age of 7 years an adequate social-communicative development and adaptive functioning in the absence of stereotypies or repetitive behaviors. Moreover, P. shows a positive psychotic symptomatology and disorganized behavior that could identify him as VEOS, although the onset was atypical depending on the age, and the psychotic symptomatology did not appear clear and explorable due to the muteness and socio-communicative closure of the child. The case of P. does not appear to align with the typical characteristics of ASD due to the late onset of a severe and rapid socio-communicative regression accompanied by symptoms of bizarre behavior suggestive of psychosis. Similarly, it does not fit the profile of VEOS due to the absence of clear and well-defined psychotic symptoms and the extremely early onset of such symptoms. Consequently, the disorder that encompasses the various characteristics of this case seems to be CDD, which demonstrates a delayed onset of socio-communicative regression along with possible psychotic symptoms, even in childhood. However, even this diagnosis does not appear to fully capture the complexity of the case, leading us to opt for a descriptive diagnosis of autism spectrum disorder with acute and late regression, in association with psychotic symptoms and a loss of primary autonomies. It seems that the symptomatology has common features among the three disorders. Each of the three diagnostic entities (ASD, VEOS, and CDD) possess identifiable and relevant characteristics, yet none of them alone provides a comprehensive picture of the clinical case.

It would be useful to think about a common spectrum disorder inserted within a psychopathological continuum between ASD and SSD, especially in those children with late-onset regression (after 4 years) and psychotic manifestations: a condition in which nuanced elements of the neurodevelopmental disorder can signal a subsequent regression in a frank autistic and psychotic-like symptomatology, even after the completion of normal evolutionary stages.

The DSM-5 is a commendable classification system for psychiatric pathologies, and its new dimensional approach enhances the ability to assess conditions that do not fit neatly into specific categories. However, this task is not always straightforward. For instance, cases like the one illustrated in this report demonstrate how certain conditions can manifest as a bridge between psychopathological dimensions. Consequently, we believe, as elucidated in the text, that it is crucial to improve the diagnostic tools in a similar way to the DSM-5, considering the continuum of pathological dimensions between neurodevelopmental and psychiatric conditions. The emphasis of this paradigm should be placed on integrating these diverse conditions rather than categorizing them in opposition to one another.

The study has several strengths, including the unique and distinctive nature of the described symptoms, the early age of onset, and the comprehensive assessment that was conducted. However, one main limitation of the study was the need for further evaluation over time to observe any potential changes in the symptomatology. This longitudinal approach is crucial for reaching a definitive diagnosis and gaining a deeper understanding of the case.

The Case Report was written following the CARE guidelines (the CARE guideline checklist is provided in the Supplementary Materials, Supplementary Figure S1). We did not provide the patient perspective as the patient had serious socio-communicative and language alterations.

As a take-home message, we want to highlight that our diagnostic difficulty reflects a clinical reality in which we cannot easily distinguish between neurodevelopmental and psychiatric aspects. Therefore, any assessment and treatment should be tailored to age, the level of neurodevelopment, and the “symptom stage.” Perhaps to better emphasize the psychopathological continuity and improve the communication of diagnosis to parents, it would be useful to conceptualize a new spectrum dimension for this nosological entity given the current division between ASD and VEOS and the previous separate diagnosis of CDD. Further studies are necessary to better understand the clinical features, causes, and efficacious treatments in this clinical manifestation.

## Data availability statement

The datasets presented in this article are not readily available because patient privacy and security are protected, according to the ethical rules of our institutions and their restriction on data sharing. Requests to access the datasets should be directed to michelangelo.diluzio@opbg.net.

## Ethics statement

The studies involving human participants were reviewed and approved by Children Hospital Bambino Gesù. Ethic Committee Name: Approval Code: 243_OPBG_2021 Approval Date: 27 October 2021. Written informed consent to participate in this study was provided by the participants' legal guardian/next of kin. Written informed consent was obtained from the individual(s), and minor(s)' legal guardian/next of kin, for the publication of any potentially identifiable images or data included in this article.

## Author contributions

MD, ML, and SG: conceptualization. MD, SG, and ML: writing—original draft preparation. LC and MP: writing—review and editing. MP, GV, and SV: supervision. All authors have read and agreed to the published version of the manuscript.

## Conflict of interest

The authors declare that the research was conducted in the absence of any commercial or financial relationships that could be construed as a potential conflict of interest.

## Publisher’s note

All claims expressed in this article are solely those of the authors and do not necessarily represent those of their affiliated organizations, or those of the publisher, the editors and the reviewers. Any product that may be evaluated in this article, or claim that may be made by its manufacturer, is not guaranteed or endorsed by the publisher.
